# Chronic furosemide administration blunts renal BOLD magnetic resonance response to an acute furosemide stimulus in patients being evaluated for renal artery revascularization

**DOI:** 10.1186/1532-429X-15-S1-P238

**Published:** 2013-01-30

**Authors:** Michael E Hall, Michael Rocco, Tim M Morgan, Craig Hamilton, Matthew Edwards, Jennifer Jordan, Justin Hurie, W Gregory Hundley

**Affiliations:** 1 Cardiology, Wake Forest School of Medicine, Winston-Salem, NC, USA; 2 Nephrology, Wake Forest School of Medicine, Winston-Salem, NC, USA; 3 Biostatistical Sciences, Wake Forest School of Medicine, Winston-Salem, NC, USA; 4 Biomedical Engineering, Wake Forest School of Medicine, Winston-Salem, NC, USA; 5 Vascular Surgery, Wake Forest School of Medicine, Winston-Salem, NC, USA

## Background

Blood Oxygen Level Dependent (BOLD) magnetic resonance (MR) is a novel imaging tool that is able to detect tissue oxygenation and has recently been utilized to evaluate renal function in patients with renal artery stenosis (RAS). Renal BOLD imaging is typically performed before and after a furosemide stimulus to assess kidney viability. Furosemide blocks the sodium-potassium-2chloride transporter, an oxygen-dependent process, in the ascending loop of Henle located in the renal medulla. Presumably, kidneys that are able to decrease oxygen consumption and increase BOLD (T2*) signal in response to furosemide would be viable and benefit from revascularization procedures. A standard dose of 20mg of intravenous (IV) furosemide is administered to evaluate renal responsiveness (increased T2* signal intensity) and viability. However, little is known about the effect of prior exposure to furosemide on the ability of BOLD MR techniques to evaluate renal function.

## Methods

We performed comprehensive MR evaluations of renal artery blood flow with phase contrast angiography and renal oxygenation with BOLD MR (1.5T Siemens Avanto) on 54 patients referred for abnormal renal arterial Doppler exams. We measured BOLD signal intensity (T2*) in the renal cortex and medulla before and after 20mg IV furosemide administration as per previously published studies. Renal function was assessed by the CKD-EPI glomerular filtration rate (GFR) calculation. Participants that take furosemide chronically abstained the morning of the MR scan.

## Results

Our study included participants with a wide range of GFR (21-136 ml/min per 1.73m2, mean 55 ± 26) and age (46-88 years, mean 69 ± 9). 54% were women, 32% were African American and 68% Caucasian, and 37% were diabetic. Home furosemide dose ranged from 0 mg/day to 160 mg/day. 60% of patients evaluated were furosemide naïve. After controlling for age, GFR, gender, race, renal artery stenosis (%), and body mass index we evaluated participants' home doses of furosemide to determine if people who are chronically administered furosemide exhibit similar changes in tissue oxygenation as those who do not receive chronic furosemide therapy. Home furosemide dose was an independent, negative predictor of renal medullary T2* response (p=0.011) in response to a standard 20mg IV furosemide challenge.

## Conclusions

These data suggest that patients who are chronically administered loop diuretics, especially those receiving large daily doses (≥ 100mg/daily), may need a different dosing strategy to accurately detect changes in renal oxygenation in response to a furosemide stimulus with BOLD MR. These findings are particularly important in this patient population because patients who are chronically administered furosemide (common in patients with RAS) may be misclassified as having nonviable kidneys due to an attenuated T2* signal intensity increases assessed with BOLD MR.

## Funding

R42 AG030248 NIH (Hundley)

**Table 1:** In a study of 54 patients evaluated for renal artery stenosis by BOLD MRI before and after furosemide administration, only race and total daily dose of lasix were independent predictors of changes in renal oxygenation.

Effect		Estimate	Standard Error	p-value
Intercept		4.8	2.1	0.57

Region	Cortex	-4.3	2.2	0.063

Kidney	Left	0.68	2.7	0.81

Gender	Men	-2.0	2.9	0.95

Race	AA	7.2	3.21	0.038

Age (years)		0.067	0.23	0.77

GFR (ml/min/ 1.73m^2)^		-0.014	0.062	0.83

Stenosis (%)		-0.087	0.062	0.076

BMI (kg/m^2)^		0.060	0.23	0.80

Lasix dose (mg/day)		-0.087	0.03	0.011

